# The Perfect Bacteriophage for Therapeutic Applications—A Quick Guide

**DOI:** 10.3390/antibiotics8030126

**Published:** 2019-08-23

**Authors:** Lucía Fernández, Diana Gutiérrez, Pilar García, Ana Rodríguez

**Affiliations:** 1 Instituto de Productos Lácteos de Asturias (IPLA-CSIC), (DairySafe Group), Paseo Río Linares s/n -Villaviciosa, 33300 Asturias, Spain; 2 Instituto de Investigación Sanitaria del Principado de Asturias (ISPA), 33011 Oviedo, Spain; 3 Laboratory of Applied Biotechnology, Department of Applied Biosciences, Faculty of Bioscience Engineering, Ghent University, 9000 Ghent, Belgium

**Keywords:** bacteriophages, biofilms, novel antimicrobials

## Abstract

The alarming spread of multiresistant infections has kick-started the quest for alternative antimicrobials. In a way, given the steady increase in untreatable infectious diseases, success in this endeavor has become a matter of life and death. Perhaps we should stop searching for an antibacterial panacea and explore a multifaceted strategy in which a wide range of compounds are available on demand depending on the specific situation. In the context of this novel tailor-made approach to combating bacterial pathogens, the once forgotten phage therapy is undergoing a revival. Indeed, the compassionate use of bacteriophages against seemingly incurable infections has been attracting a lot of media attention lately. However, in order to take full advantage of this strategy, bacteria’s natural predators must be taken from their environment and then carefully selected to suit our needs. In this review, we have explored the vast literature regarding phage isolation and characterization for therapeutic purposes, paying special attention to the most recent studies, in search of findings that hint at the most efficient strategies to identify suitable candidates. From this information, we will list and discuss the traits that, at the moment, are considered particularly valuable in phages destined for antimicrobial therapy applications. Due to the growing importance given to biofilms in the context of bacterial infections, we will dedicate a specific section to those characteristics that indicate the suitability of a bacteriophage as an antibiofilm agent. Overall, the objective is not just to have a large collection of phages, but to have the best possible candidates to guarantee elimination of the target pathogens.

## 1. Phage Therapy: A Major Comeback with Minor Setbacks

The existence of viruses that can infect and kill bacterial cells, known as bacteriophages (or phages), was discovered almost simultaneously by Frederick Twort and Félix d’Herelle about one century ago, but it is the latter who has been credited with introducing the concept of using these entities as antimicrobial agents [1,2]. Indeed, the French-Canadian microbiologist pioneered the so-called phage therapy and used phages to treat patients with various infectious diseases [3]. Since that time, the utilization of phages for therapeutic applications has remained common practice in Eastern Europe, saving countless human lives [4]. Conversely, in other parts of the world, the introduction of antibiotics pushed this anti-infective strategy out of center stage for many decades. After all, antibiotics were cheaper to manufacture and exhibited a broader spectrum of activity, thereby eliminating the need to identify the etiological agent prior to treatment prescription. However, with time, overuse and misuse of antibiotics became widespread in different fields, such as human and veterinary medicine or agriculture. In this context, antibiotics exerted the selective pressure that resulted in the spread of resistance markers and led up to the current antimicrobial resistance crisis [5]. Alarmingly, the number of deaths associated with bacterial infections is rising and threatens to reach levels not seen since the preantibiotic era. The reason for this is that more and more bacterial strains are acquiring multiresistance and are, consequently, able to survive treatment with most, if not all, antibiotics commonly used in the clinic. Given the severity of such a scenario, besides curtailing unnecessary antibiotic use, the scientific community has started a race to find as many alternatives to conventional antibiotics as possible [6]. Among the many options being explored, the revival of phage therapy presents itself as an attractive strategy to substitute or complement other therapies [7]. This is especially the case because phages, due to their unique mechanism of action, exhibit some notable differences compared to other types of antimicrobials, some of which are particularly advantageous. These positive attributes include their natural origin, lack of toxicity for humans or nontarget microbes (being harmless to the normal microbiota), and their effectiveness against antibiotic-resistant bacteria, amongst others. On the downside, phage therapy generally requires identification of the target pathogen and phage resistance development remains a possibility, although it is not usually as easily transferable to other microorganisms as antibiotic resistance determinants. Moreover, resistance could be easily overcome by using combinations of different phages (phage cocktails) instead of a single phage for therapeutic applications [8].

Nonetheless, not everything is rosy on the way toward normalizing the use of bacteriophages against unwanted bacteria in the context of human medicine. Most notably, it will be paramount to convince both the general public and the competent authorities that these antimicrobials are safe for human health and the environment, as well as provide undeniable proof of their efficacy in clinical settings. Indeed, these are two key aspects in order to surmount the current regulatory constraints that limit the use of bacterial viruses for infection treatment in humans. Until relatively recently, most evidence regarding the effectiveness of phage therapy was the result of direct use in patients, but well-organized and reproducible data will be essential in order to comply with the requirements set out for the approval of medicinal products [9]. In this regard, the growing number of completed clinical trials will certainly be invaluable. On the other hand, the growing utilization of phages as biocontrol agents for the disinfection of inert surfaces may also help to pave the way toward regulatory approval [10]. As a matter of fact, the relevant authorities in different countries (USA, Israel, Canada, Switzerland, Australia, New Zealand, and the Netherlands) have already approved this type of applications against several pathogens in the context of the food industry [11,12]. For example, there are several commercially available products to be used against various food pathogens, such as *Listeria monocytogenes*, *Salmonella*, or *Escherichia coli*, as surface disinfectants or processing aids. Indeed, the FDA has recognized some phage-based formulations as “generally regarded as safe” (GRAS) as food additives. Regarding medicinal use, phage therapy is now under the scrutiny of government agencies, which are trying to determine the most adequate regulatory framework considering the special characteristics of phages. In the meantime, patients in many countries can have access to this treatment as compassionate use [13]. One of the major challenges of regulating phage approval is the diversity of phages that will be necessary to successfully implement this therapeutic strategy. Indeed, in order to harness the full potential of phage therapy and its adaptability to changes in pathogenic strains, there should be room for making changes to phage-based formulations without the need for a lengthy and costly approval process. In addition to regulatory hurdles, bringing phage therapy to the market also has the problem of not being very attractive for pharmaceutical companies. To some extent, this is a consequence of their natural origin, as phages cannot be patented. Also, their use is aimed to be used as an acute course of treatment rather than for chronic administration, which obviously means that they would not be as profitable as other medicines. However, the relevant authorities can help by designing an affordable and straightforward pathway for regulatory compliance that might perhaps encourage the participation of smaller specialized companies.

**Figure 1:**
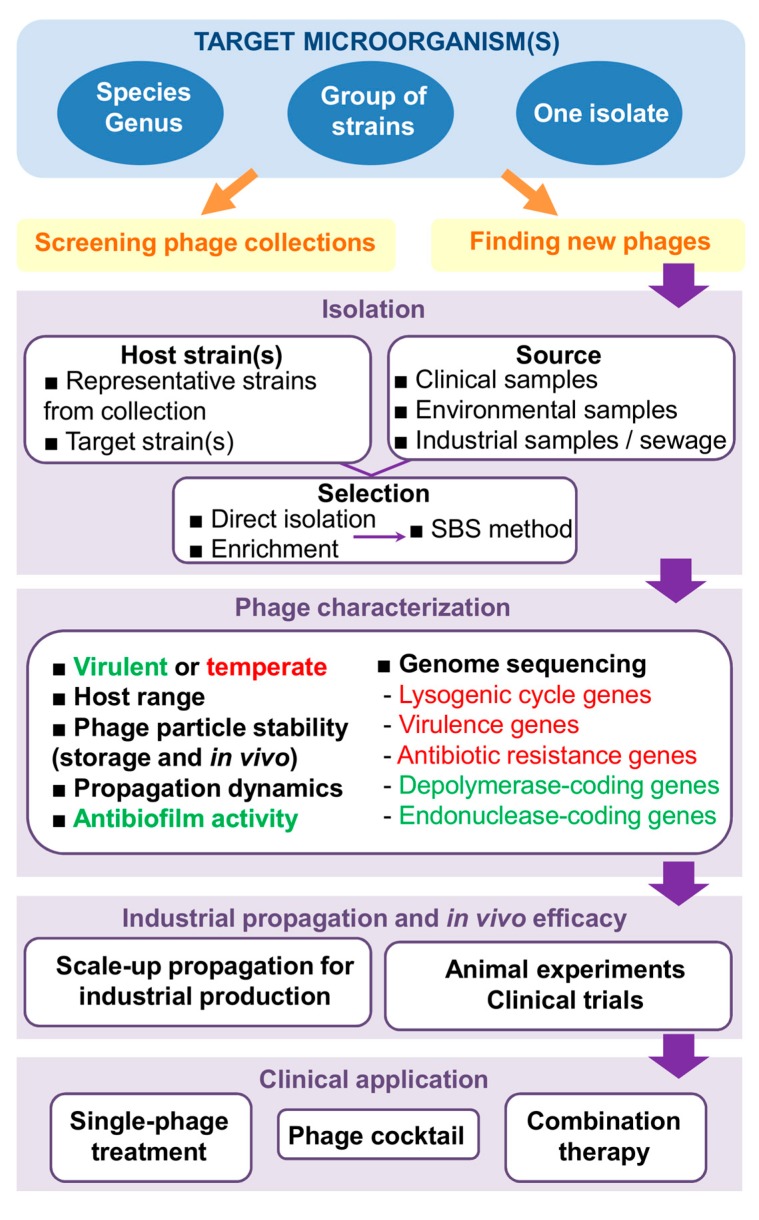
Steps involved in the development of phage therapy strategies. In the phage characterization step, text in green and red respectively correspond to desirable and undesirable characteristics in a phage for therapeutic applications. Abbreviations: SBS method, step-by-step method.

Overall, it is apparent that phages are getting closer and closer to becoming a mainstream antimicrobial treatment option, especially in the case of difficult-to-cure multiresistant infections. However, in order to get the most out of phage therapy, we will need to have an arsenal of well-chosen effective and safe bacteriophages at our disposal. This will involve a number of steps that, if not carefully planned, may hinder the successful development and subsequent implementation of this strategy (Figure 1). In order to maximize speed and minimize costs, it is necessary to establish clear guidelines indicating where and how to look for suitable therapeutic phages, as well as define the most valuable traits in the identified candidates.

## 2. In Search of New Phages

One of the main advantages of using bacteriophages as antimicrobials is their sheer quantity and diversity. As a matter of fact, even without improvement by genetic engineering, nature represents an almost limitless source of new phage variants. It is well known that phages are the most abundant biological entities on earth (about 10^31^ phages in total) [14]. On top of that, bacterial viruses evolve at a dizzying rate and are, as a result, highly diverse. Indeed, there is evidence indicating that bacteriophages outpace their hosts in the coevolutionary race [15]. Now, this all sounds like good news from the perspective of the antimicrobial development pipeline until coming to the realization that finding the right phages for our purpose among this huge set of candidates is quite the gargantuan task. Nevertheless, it is also worth noting that vast phage collections are already available in different research centers and universities around the globe. As a result, this would be a good place to start once the target microbe is identified.

It is obviously impossible to screen for phages everywhere, so a major step consists in narrowing down the most probable reservoirs of viruses that infect and kill our target microbe. As is always the case with predators, these reservoirs are likely to coincide with the main habitats of their prey, that is, the target pathogen. In that sense, d’Hérelle suggested that the best source of bacterial viruses for antimicrobial applications are samples from patients who are recovering or who have just recovered from an infection [16]. Indeed, he first remarked the existence of “bacteria eaters” in stool samples of patients recovering from bacillary dysentery [1]. Similarly, phages against enterohemorrhagic *E. coli* (EHEC) O157:H7 were found in fecal samples from human patients and cattle [17]. Additionally, phages infecting *Cutibacterium acnes* (formerly *Propionibacterium acnes*) and *Actinobacillus* have respectively been isolated from human skin and dental plaque [18,19]. Given the prevalence of human pathogenic bacteria in infected patients, another good reservoir of phages infecting these microorganisms is wastewater from hospitals [20]. If clinical samples are not available, good results have also been obtained with sewage water, which has actually become a frequent starting point for phage hunts [21]. In the case of microbes with high prevalence in diverse ecological niches, different environmental sources like river or stream water have also been the place to isolate new bacteriophages [22,23]. Additionally, drinking water was the source of phages infecting *Enterococcus* and *Staphylococcus* [24], while silage from dairy farms proved a good source of listeriaphages [25].

In some cases, the phage titer in these reservoirs will be high enough to allow direct isolation of the virus after filtration of the sample; for example, by visualization of lysis plaques on plates containing the host (double layer assay). However, this is not always the case and enrichment steps are often required to detect the presence of the phage. Frequently, it is a good idea to collect large-volume samples in order to increase the chances of phage isolation, especially when dealing with samples taken from environmental sources. It must also be noted that some phages form very small plaques on a bacterial lawn, which would hinder their identification by the double layer method. For this reason, if no plaques are observed during the isolation step, it might be worth repeating the process but using techniques that increase plaque size, such as addition of subinhibitory concentrations of antibiotics [26,27] or other compounds like sodium thiosulfate, ferric ammonium citrate or 2,3,5-triphenyltetrazolium [28].

Another important aspect in the quest to find new phages with antimicrobial potential lies in the selection of the most appropriate host strain or strains. However, this choice will also depend on the specific goal of the search. Thus, if the objective is the identification of phages against as many strains of a given pathogen as possible, multihost selection protocols should be used. Additionally, the host strains should preferably be very varied in terms of their characteristics and origin so that they are representative of the species. In this sense, Casey et al. [29] recommend using bacteria reference collections for phage identification, as they offer a good representation of the intraspecies diversity, together with strains of clinical relevance. However, in some cases, there may be a specific target strain or group of strains that do not seem to be susceptible to the phages available in different collections. Then, it will be necessary to carry out screenings using these strains as selection hosts. Nonetheless, if finding a new virulent phage that infects our target strain proves to be very difficult, it is possible to carry out an adaptation strategy in which coevolution rounds may allow the selection of a phage variant that can effectively infect and kill the pathogenic strain. This protocol is called the step-by-step (SBS) method [30]. The phenomenon of local host adaptation also plays a role in the identification of new phages [31]. Indeed, phages are known to co-evolve very closely with their host strains. As a result, virulent phages from a given reservoir or geographic location are oftentimes more effective at targeting strains from that same milieu.

## 3. Desirable Traits in Phages with Antimicrobial Potential

### 3.1. Specific but Not Too Specific

One of the major advantages of bacteriophages as antimicrobials is their specificity. A consequence of this characteristic is that they are not only innocuous to eukaryotic cells, but also to all prokaryotic cells outside their host range. This would ensure that the normal microbiota remains intact during therapy, which in turn would be expected to help prevent secondary infections and probably accelerate recovery. Indeed, there is growing proof that the microorganisms inhabiting our bodies play very important roles in our overall health. Antibiotic treatment, by contrast, can severely affect our normal microbiota due to their more general, broad-range action, which often results in serious side effects [32]. Another benefit of this specific action is that we can technically try to identify viruses that only infect certain strains within a bacterial species, distinguishing, for example, between pathogenic and nonpathogenic isolates. Again, some microbes can potentially be helpful or harmful to their human hosts depending on the presence of sometimes only a few genes that turn them from friend to foe. As a result, it would be ideal to develop antimicrobials that can differentiate between commensal and pathogenic strains of the same species.

Notwithstanding the incredible target precision that can be achieved with phage therapy, it is generally preferable to isolate viruses able to attack a wide range of strains within a bacterial species. In some cases, it may also be desirable that a given phage can infect different pathogenic species within a genus. Staphylococcal phages are a good example of this, with some of them infecting the two opportunistic pathogens *Staphylococcus aureus* and *Staphylococcus epidermidis*, such as myophages phiIPLA-RODI and phiIPLA-C1C [33]. The polyvalent nature of staphylophages is also useful since it allows carrying out phage propagation on nonpathogenic species, such as *Staphylococcus xylosus* [34]. Remarkably, there are phages capable of infecting different genera within the *Enterobacteriaceae* family, including *E. coli* O157:H7, *Salmonella enterica* ser. Paratyphi, and *Shigella dysenteriae* [35].

At first glance, it would appear that having a broad host range would be a synonym of success for a bacteriophage, as it would increase its likelihood of encountering a susceptible bacterial cell, which is necessary for survival of the phage population [36]. However, this hypothesis is not supported by the available experimental data. Thus, the ability of a given phage to infect and multiply inside a host depends on factors from both prey and predator. As mentioned previously, some studies have observed a very well-tuned coevolution, leading to local specificity in phage–host interactions [31]. Indeed, there is evidence that greater specialization can be linked to increased infection efficacy and the other way around, which suggests that there is an evolutionary compromise between these two properties [37,38]. Therefore, while it is possible to broaden the host range of a given phage, this might lead to a “weaker virus” that cannot propagate as efficiently.

In any case, a bacteriophage used for therapeutic applications should at the very least be able to infect the different variations of the pathogen population inside the patient. As this is not always possible with single-phage preparations, the use of a phage cocktail, if available, is always a better option. Interestingly, study of the coexistence dynamics between the cyanobacterium *Prochlorococcus* and ten cyanophages revealed that acquisition of resistance to one phage sometimes resulted in enhanced infection by other phages [39]. In some cases, the authors could demonstrate that this was due to the increased adsorption of “the other phages” to the phage-resistant mutants than to the original bacterial strain. If this phenomenon is also observed in therapeutic phages, it would be useful in the context of phage cocktail administration, as it would manifest itself as a synergic rather than an additive effect between the different viruses. Identification of the potential phage receptors in the host is an important aspect of designing a phage cocktail. Evidently, it would be better to have experimental evidence of the nature of the receptor for a specific phage. However, if this information is not available, there are now resources to predict potential receptor molecules based on the phage genome data and information from literature or databases [40,41]. Indeed, it would help to avoid combining phages that can potentially exhibit cross-resistance. Moreover, it would be preferable to select phages whose target receptors are essential for bacterial fitness or virulence as the likelihood of phage resistance selection will be lower or, at the very least, might lead to lesser pathogenicity. Furthermore, if there is receptor modification, the probability of phage variants that can infect the altered receptor are much higher than they are in cases of receptor loss, a possibility with nonessential receptor molecules [42]. Indeed, infectivity evolution in phages during therapeutic applications should be studied more in depth. Especially, because it provides a unique opportunity of using an evolving antimicrobial that can potentially overcome by itself bacterial resistance mechanisms during therapy [42].

Another strategy for limiting phage resistance is the selection of phages harboring modified nucleotides, like archeosine or N6-(1-acetamido)-adenine, in their genomes. This would make the viral DNA resistant to the activity of many restriction endonucleases, one of bacteria’s natural antiphage defense systems [43]. A hint of this trait can be found during genome sequencing through the identification of genes involved in the biosynthesis of these nucleotides. For instance, recent studies showed that siphophages Vid5 and BRET, which respectively infect the enterobacteria *Pantoea agglomerans* and *E. coli*, carry archeosine biosynthesis genes [44,45].

### 3.2. Virulent Phages Only, Please

In addition to the lytic cycle, which ends up with the death of the bacterial cell, some bacteriophages can also undergo the so-called lysogenic cycle. In this case, the viral genome integrates into and replicates with the bacterial chromosome. The viral genetic material, known as prophage, can stay in this state for generations until an environmental signal turns on the machinery leading to its excision from the bacterial genome and kick-starts the lytic cycle. This type of phage is called temperate and they are known to actively contribute to the phenomenon of horizontal gene transfer, one of the main mechanisms involved in the spread of antimicrobial resistance and virulence genes. As a result, temperate phages are not considered good candidates for phage therapy. In contrast, phages that can only undergo the lytic cycle, or virulent phages, are ideal for this purpose, as their multiplication is almost always followed by lysis of the infected cell. These phages are also sometimes called “professionally lytic” [46]. During the phage selection process, virulent and temperate phages can tentatively be differentiated as they respectively produce clear and turbid plaques on a bacterial lawn.

In some cases, phages that can infect and kill the target organism, especially if it is a specific strain, are scarce and utilization of virulent derivatives from temperate phages is required. This can be achieved by selecting or constructing mutant phage variants through different protocols. For example, phage deletion mutants can be easily isolated by exposing the phage particles to several rounds of a chelating agent such as ethylenediaminetetraacetic acid (EDTA), sodium citrate, or sodium pyrophosphate [47]. Chelating agents exert a destabilizing effect on the phage particles by binding the cations located on their surface, which results in conformational defects and, ultimately, in viability loss. However, viral particles carrying deletions in their genome are smaller and can retain their conformation and remain viable under these conditions. From the selected deletion mutants, it would be then possible to identify the virulent variants, as they would produce clear instead of turbid plaques. Alternatively, phages can be genetically manipulated to obtain a virulent variant by removing genes involved in lysogeny. Indeed, a cocktail containing engineered phages successfully eliminated an infection by a drug-resistant strain of *Mycobacterium abscessus* from a cystic fibrosis patient [48]. However, the use of genetically modified organisms is still shrouded in controversy and is not even a viable option in many countries.

Nevertheless, strictly virulent phages are not completely free from the danger of facilitating transfer of antibiotic resistance genes. For instance, Keen et al. [49] recently described the existence of “superspreader” virulent phages, which do not carry endonucleases in their genome and release the bacterial DNA to the environment. This would make it possible that this now extracellular DNA can enter other microorganisms by natural transformation and potentially spread resistance or virulence markers. In fact, this study reported that addition of the phage to cocultures of a kanamycin-sensitive *Bacillus* sp. and kanamycin-resistant *E. coli* strains led to a 1000-fold increase in transfer of the antibiotic resistance marker to *Bacillus*. This would not be a desirable situation so it would be important to ensure that a phage with therapeutic potential does degrade the bacterial chromosome prior to cell lysis.

Once confirmed that a candidate phage is strictly virulent and not a superspreader, the next step is to determine if it can propagate efficiently both in vitro and in vivo. First, it is necessary to study propagation under in vitro conditions, for example, by performing a one-step growth curve. This technique requires achieving the synchronous infection of all bacterial cells in a population in order to then monitor how the number of extracellular and intracellular viable phage particles changes throughout the lytic cycle. This data will allow calculation of valuable propagation parameters, such as burst size (average number of new phage particles released per infected cell) and latent period (part of the lytic cycle between virion attachment to the cell and start of new phage particle release). In some cases, a shorter latent period has been associated with the possession of DNA-dependent RNA polymerases in the phage genome [50]. Theoretically, this protein might shut down host transcription and increase the efficiency of the phage replication cycle, although this phenomenon has not been supported by experimental evidence yet. After confirming that the phage can propagate under in vitro conditions, which is essential for phage production, in vivo experiments should be performed to confirm that the virus can also multiply effectively at the infection site if the target host is present. This ability to self-propagate during treatment is undoubtedly a major advantage compared to other types of antimicrobials, whose concentration invariably decreases after administration to the patient. 

### 3.3. Safe for Human Health and the Environment

Following the isolation of a phage with therapeutic potential, it is necessary to perform genome sequencing and analysis. Thanks to the advances made in this field, this has become an easy and affordable task that can actually save us a lot of time and money on additional experiments. On the one hand, examination of the phage genome can confirm that there are no genes involved in lysogeny, such as the lytic cycle repressor, integrases or site-specific recombinases. Identification of genes encoding potential endonucleases can also suggest that it is not a “superspreader”. On top of that, genomic analysis is the single most reliable way to know whether the phage genome encodes toxins or antimicrobial resistance determinants. Indeed, if there are any such genes, the phage should never be considered for therapeutic applications. If possible, it is also preferable to choose a propagation host (a so-called surrogate strain) that does not carry prophages or virulence/antibiotic resistance markers [51].

Besides not being carriers of “bad genes”, bacteriophages must be innocuous when administered to the patients. As mentioned above, one key aspect of phages as therapeutics is the fact that they do not infect nontarget bacteria, leaving the normal microbiota largely undisturbed after therapy. Regarding their safety for the patient, all clinical trials so far indicate that they do not exhibit significant toxicity. Nonetheless, it is worth noting that phages need to be well purified in order to remove toxic substances before administration. A clear example of this is endotoxin, which could lead to serious side-effects if not thoroughly removed from the phage suspension. The importance of endotoxin removal depends, however, on the type of application. For example, it is essential for intravenous administration, while purification does not need to be so strict for topical or oral administration to the patient [51,52]. In that sense, it is also important to consider the potential effect of widespread lysis of the target bacteria inside the patient. It is worth noting, however, that studies in which filtered lysates were administered directly to patients without further purification led to mild or, most frequently, no symptoms [53]. Overall, the available data suggests that the doses required for bacteriophages to trigger toxicity exceed the effective concentrations necessary for their application. This holds true not only for the administered doses but also considering the propagation inside the patient. As such, phages typically exhibit a high therapeutic index, a quantitative measurement of the relative safety of a drug, which is obtained as the ratio between the dose leading to toxicity and the amount that displays antimicrobial activity.

Due to the relative simplicity and lack of diversity of bacteriophages in terms of their chemical composition, basically consisting of proteins and nucleic acids, their potential interactions with the immune system are relatively easy to study and predict in stark contrast to other types of antimicrobials. Given that the outward structure of the viral particle consists basically of proteins, phages can be immunogenic. This issue would be especially problematic when intravenous (i.v.) administration is required, as it could potentially lead to an anaphylactic response. However, i.v. administration of phages has been performed repeatedly without observing major negative side effects [52].

Widespread phage application as an antimicrobial also poses questions regarding its potential impact on natural environments [54]. Indeed, it is very important to assess the risk of altering the composition of natural microbial communities due to the release of phages from clinical, veterinary or agricultural applications. These changes could be deleterious to ecosystems as they might alter nutrient cycling. Given the structure of these communities, an impact of phages on soil ecological dynamics (phage to bacteria ratios of 1:1) would be more expected than on aquatic communities (phage to bacteria ratios of 1:100). However, this disruptive effect would be expected to be much lower than that of antibiotics given the specificity of bacteriophage activity.

A key aspect to ensure the safety of phages aimed for therapeutic applications is the development of well-defined bacteriophage production and quality control protocols that allow manufacturing of pharmaceutical-grade phage products [55]. These methods should maximize phage yield in the production stage followed by highly effective purification steps [55,56]. Thus, while satisfying health and environmental concerns should be a priority, the economic viability of industrial phage production must also be taken into account. All of these steps are also very important in order to achieve regulatory approval of phage-based formulations [57].

### 3.4. Stability

As mentioned above, phage particles consist of a nucleic acid molecule inside a proteinaceous envelope. As such, they are much more complex and labile compounds than other antimicrobials. This fact makes them unstable outside a specific range of environmental conditions (temperature, pH, UV-light, salt concentrations, proteases). Thorough analysis of phage particle stability is, therefore, an integral part of the phage characterization process. For example, a very effective virulent phage that has a very narrow stability range may not be well suited for therapy. Indeed, the viral particles must be able to withstand the production, storage and administration stages in sufficient numbers to reach their target. However, the conditions that allow phage stability maintenance vary enormously depending on the specific phage [58]. In fact, it appears that neither family nor close structural similarity are good predictors of stability range, although tailed phages generally seem to be the most stable under adverse environmental conditions [59].

After propagation, the bacteriophage should remain viable and infective throughout storage. As mentioned above, specific storage conditions have to be tested for each phage, as they may vary depending on its characteristics. For example, different storage temperatures are better suited for different phages [59]. Thus, while some are more stable at temperatures around 4 °C, others must be kept at freezing temperatures of −20 °C or even −80 °C. In contrast, some phages exhibit very good stability at room temperature or even 37 °C. Another important factor is the format for storing the phage particles. For instance, some phages remain viable for longer periods of time when stored in a liquid (which can be growth medium or a buffer). An alternative possibility is storing the phage particles in a dry powder, which can be produced by different techniques such as lyophilization (freeze drying) or spray drying. In this case, it is necessary to consider that the drying process itself may be deleterious for the viral particles, which might lead to a considerable titer loss prior to storage [22,60]. Sometimes, preservation in both liquid and dry form can be improved by adding different stabilizing agents, such as skim milk, glycerol, trehalose, or sorbitol [61]. Alternatively, the phages can be kept as a nucleic acid inside infected cells, which can then be stored frozen at −80 °C [62]. The lytic cycle would continue as soon as bacterial growth is resumed. However, González-Menéndez et al. [60] showed that this technique does not always improve phage particle stability compared to storage of the naked phages. Some studies have also shown that phage encapsulation in nanovesicles can enhance stability by protecting the particles from environmental factors, although a study showed that this improvement was only noticeable during short-term but not during long-term storage [63].

In order to be a good candidate for phage therapy, the viral particles must be stable not only during the production and storage stages, but also during its application, that is, it has to remain infective from the moment of administration to the patient until it reaches the target pathogen. In that context, the characteristics of the phage and the type of formulation used will depend on the administration route and target organs. With regards to temperature, for example, the phage should be able to withstand the body temperature for long enough to reach its target host. Additionally, if application is to be carried out by topical administration, it would be desirable that the phage is not exceedingly sensitive to UV-light exposure, so that it is not inactivated before infecting the bacterial cells. If treatment is aimed at respiratory infections, the phage particles should be able to withstand techniques used for the preparation of aerosols, such as freeze- or spray-drying [64,65,66]. In turn, the ability to withstand an acidic pH will be very important if the phage is to be administered by the oral route.

As was the case with storage, encapsulation of the phage particles can also be helpful in order to improve stability during treatment. For example, by facilitating skin absorption of the product so that the phage can reach deeper layers of the epidermis, or by protecting the phage particles from the acid in the stomach or from bile salts [67]. Encapsulation can also be suitable for inhalation of a phage formulation that has to reach the lungs. For instance, a study by Singla et al. [68] showed that phages in liposomes were more effective than nonencapsulated phages for treating pneumonia caused by *Klebsiella pneumoniae*. Additionally, encapsulated phages displayed a greater ability to enter eukaryotic cells than their nonencapsulated counterparts [69]. This indicates that they have greater potential for reaching intracellular pathogens. Recent work by Nobrega et al. [70] demonstrated that improved phage stability during oral administration could be achieved by genetically engineering the phage particles of the *E. coli* phage T7 to display lipids on their surface. The authors proposed this strategy as a good alternative to encapsulation. This high stability is very important as otherwise, oral administration of phages requires very high phage titers (≥10^11^ PFU/mL) so that at least a dose of 10^6^ PFU can reach the intestine.

Topical application of phages is the preferred form of treatment for various skin infections, ranging from acne to serious burn wound infections. In these cases, the phage will typically be applied as a cream. Good results for phage stability in this format have been obtained so far. For example, phages against *C. acnes* remained viable for 90 days in a semisolid preparation if stored protected from light and at 4 °C [18]. In the case of the staphylococcal phage K, stability for several days could be achieved with a cream stored at room temperature, and the results were improved when using an oil-in-water nanoemulsion [71]. The authors hypothesized that this enhanced stability may be the result of electrostatic forces [72]. Additionally, the nanoemulsion may lead to decreased electrostatic repulsion between the negatively charged surfaces of the phage particle and the cell. In the case of products aimed at treating burn wound infections, Merabishvili et al. [73] examined the stability of phages against the main causative agents (*P. aeruginosa, A. baumanii, S. aureus*) in different antibiotic-containing formulations, including creams, ointments, and hydrogels. The results of this work indicate that the pH of the product is the most limiting factor for phage stability.

Finally, the bacteriophage must be able to resist the immune response of the patient. However, it seems that a very high dose of phage particles, much higher than that recommended for treatment, is necessary in order to elicit a significant immune response [74]. According to a study by Majewska et al. [74], this response is higher if the phage is applied by subcutaneous injection rather than by oral administration. This lack of a very intense response by the immune system is probably the result of continuous exposure to phages which are part of the human microbiome [75]. In any case, definitively showing that phages can remain viable and be effective will come from further studies in animal models and clinical trials. It is also important to make sure that the phage particles are not inactivated by neutralizing antibodies. However, a humoral immune response would be more of a problem when the phages are administered parenterally rather than after oral or topical application [76]. Moreover, antibody production dynamics would be expected to occur more slowly than pathogen elimination by the phage and, as a result, phage neutralization by the immune system should not constitute a problem during acute infection therapy. Additionally, PEGylation of the phage particles, that is, addition of monomethoxy-polyethylene glycol (mPEG), is known to reduce their immunogenicity and result in lesser levels of cytokine production in mice [77]. Nonetheless, these responses still must be studied in more depth. 

### 3.5. Antibiofilm Potential

**Figure 2:**
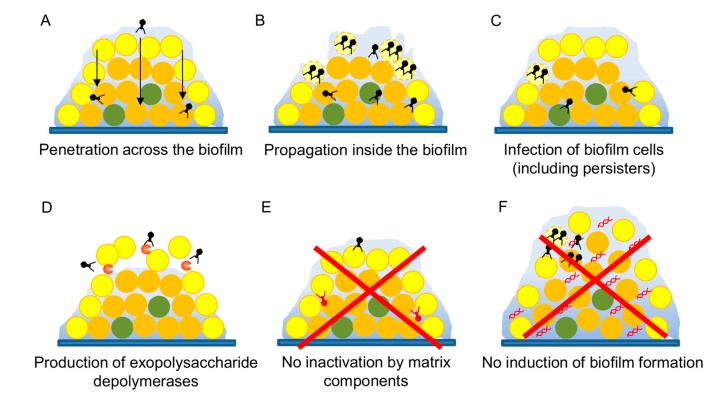
Analysis of the antibiofilm potential of a candidate bacteriophage.

Biofilms are the most common lifestyle of bacteria in both natural and artificial environments, including inside the human body. As a result, it is now known that biofilms contribute to bacterial pathogenicity, especially in the case of chronic infections [78]. These multicellular structures allow the bacterial cells to survive under normally lethal conditions, resisting challenges by the immune system or antimicrobial compounds thanks to a combination of multiple mechanisms [79]. This makes biofilms very difficult to eliminate, and one of the major problems that need to be tackled in order to combat infections. In that sense, bacteriophages may, given their special characteristics, provide an interesting alternative to conventional disinfectants or antibiotics [80,81]. However, it is necessary to demonstrate that a given phage is a useful antibiofilm agent. Several studies have already shown that phage treatment of preformed biofilms can successfully reduce the amount of total attached biomass as well as the number of viable bacterial cells attached to the surface. For instance, phage phiIPLA-RODI, which infects different staphylococcal species, can successfully kill *S. aureus* cells from preformed single-species and multispecies biofilms [33,82]. A further example was provided by Khalifa et al. [83], who showed that an enterococcal phage could eliminate in vitro biofilms and prevent infection by *Enterococcus faecalis* in an ex vivo model of root canal infection. Likewise, phages have been used to kill biofilm cells from numerous important pathogens such as *P. aeruginosa* [84], *A. baumanii* [85], and uropathogenic *E. coli* [86], amongst others. Also, some studies have demonstrated that phages can move across these complex microbial populations (Figure 2A) and propagate if a suitable host is present (Figure 2B) [87,88]. Remarkably, phages also seem to be able to infect the infamous persister cells (Figure 2C) that are known to contribute to recalcitrant infections, even if they cannot proceed with the lytic cycle until bacterial growth is resumed [89].

An interesting feature of some phages is the possession of genes coding for exopolysaccharide depolymerases (Figure 2D) that can degrade the polysaccharidic component of the extracellular matrix of biofilms facilitating biofilm dispersion and access of the phage into the deeper layers of this structure [90,91]. It is clear that including at least one such phage in a therapeutic cocktail would be of help to all the different phages. Similarly, production by phage J8-65 of a colinidase, which targets polysaccharides in the cell envelope, was responsible for synergy between this phage and T7 in a temperature- and media-dependent manner [92]. Alternatively, a bacteriophage of interest could be genetically modified to encode a depolymerase in its genome, as Lu and Collins [93] demonstrated in their proof-of-principle work with phage T7. Although this strategy would be very controversial at the moment, if engineered phages become more widely accepted for therapy applications, this would open the door for modification with other types of genes that may control biofilm development. A very interesting example was provided by Pei et al. [94] who cloned a quorum-quenching enzyme in the genome of T7 bacteriophages. While phage degradation of the extracellular matrix is a very desirable outcome, phage inactivation by matrix components should be avoided (Figure 2E).

Another important aspect to examine is whether a given phage can potentially promote biofilm formation of the target pathogen (Figure 2F). In this sense, there are examples showing that both biofilm enhancement and inhibition are possible depending on the phage–host pair, although studies concerning the impact of virulent phages have been so far outnumbered by those focusing on temperate viruses [95]. For example, different studies have shown that addition of some virulent phages leads to increased biofilm formation in strains of *S. aureus*, *P. aeruginosa*, *Salmonella*, and *Vibrio anguillarum* [96,97,98]. In some cases, such as *P. aeruginosa*, it is known that the phenotype is due to the selection of phage-resistant mutants with better biofilm-forming ability [97]. This could be solved by application of a phage cocktail, as selection of mutants against all phages in the cocktail would be less likely. In *V. anguillarum*, phage KVP40 promotes increased cell aggregation [98]. In contrast, accumulation of extracellular DNA was responsible for increased attached biomass upon low-level predation of *S. aureus* by phage phiIPLA-RODI [96]. Nonetheless, it is worth emphasizing the need to understand the specific mechanisms underlying biofilm increase by phages in order to take action to reduce or completely eliminate this undesirable phenomenon.

## 4. Conclusions

In the midst of a nearly apocalyptic scenario regarding our ability to cure infectious diseases, phage therapy seems like a saving grace. Perhaps not a perfect strategy by itself, it certainly is a good supplement to other therapies as part of a vast antimicrobial arsenal. Their specificity also fits very nicely within the tendency toward a more personalized approach to human medicine, especially when it comes to treatment strategies. However, it is paramount to have as much information as possible before phage therapy use in the clinic is generalized. This will hopefully prevent some of the errors made with antibiotics. Additionally, it is very important to have a crystal-clear view of the characteristics that make a bacteriophage a good candidate for therapeutic use. Once it is established where and how the search for new phages will be performed, careful analysis of the isolated phage should ensue. Perhaps the single most important trait is the selection of strictly lytic phages, while temperate phages should be either modified or discarded outright. In addition, phages should not carry virulence or antibiotic resistance genes in their genomes and preferably produce endonucleases that degrade the host chromosome prior to cell lysis, thereby preventing spread of bacterial genes to other microbes by natural transformation. Clearly, bacteriophages will never attain the spectrum of action of antibiotics, if only because of their particular multistep mechanism of bacterial killing, the lytic cycle, which involves several specific interactions at the molecular level. This may not necessarily be a drawback, as phage specificity also allows for tightly controlled killing of pathogenic targets without damaging eukaryotic cells or nontarget microbes. This feature is very important given the increasingly recognized role of the microbiota in human health. Another aspect that requires attention during phage selection is phage particle stability under different conditions, especially those occurring during storage and, above all, during treatment. Nonetheless, strategies that allow prolonged stability such as encapsulation should be also developed further. Last but not least, the interactions between phages and microbial biofilms should be analyzed in depth in order to maximize the potential use of bacterial viruses to reduce or eliminate these structures. Indeed, this might be a key step in the fight against chronic, recalcitrant infections that do not respond to other types of antimicrobial therapy. Overall, phage therapy seems like a viable, promising strategy that might play a major role in the antibacterial regimes of the future, perhaps even the near future. Nonetheless, further research is still necessary to fill in the gaps that will enable using this strategy while maximizing both efficacy and safety.
